# Interpretable Machine Learning of Amino Acid Patterns in Proteins: A Statistical Ensemble Approach

**DOI:** 10.1021/acs.jctc.3c00383

**Published:** 2023-08-08

**Authors:** Anna Braghetto, Enzo Orlandini, Marco Baiesi

**Affiliations:** † Department of Physics and Astronomy, University of Padova, Via Marzolo 8, 35131 Padua, Italy; ‡ INFN, Sezione di Padova, Via Marzolo 8, 35131 Padua, Italy

## Abstract

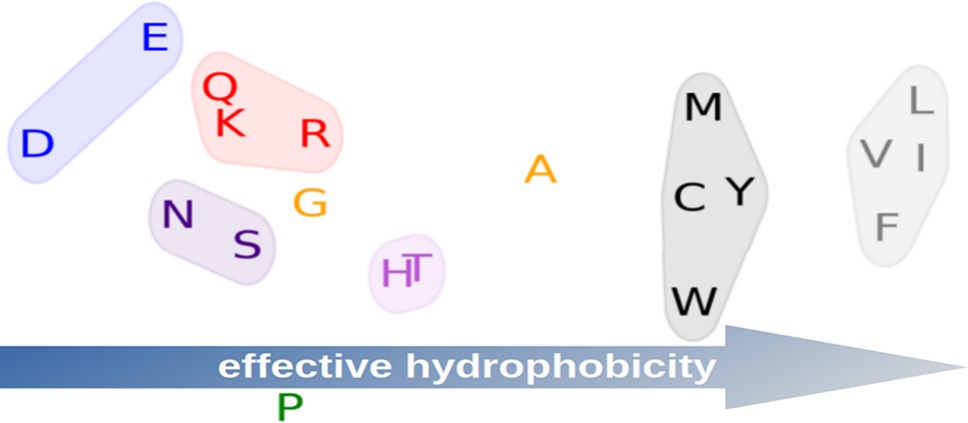

Explainable and interpretable unsupervised machine learning helps one to understand the underlying structure of data. We introduce an ensemble analysis of machine learning models to consolidate their interpretation. Its application shows that restricted Boltzmann machines compress consistently into a few bits the information stored in a sequence of five amino acids at the start or end of α-helices or β-sheets. The weights learned by the machines reveal unexpected properties of the amino acids and the secondary structure of proteins: (i) His and Thr have a negligible contribution to the amphiphilic pattern of α-helices; (ii) there is a class of α-helices particularly rich in Ala at their end; (iii) Pro occupies most often slots otherwise occupied by polar or charged amino acids, and its presence at the start of helices is relevant; (iv) Glu and especially Asp on one side and Val, Leu, Iso, and Phe on the other display the strongest tendency to mark amphiphilic patterns, i.e., extreme values of an *effective hydrophobicity*, though they are not the most powerful (non)hydrophobic amino acids.

## 1 Introduction

Various machine learning (ML) methods are applied to proteins.^1−25^ For example, outstanding advancements have shown how ML can boost the prediction of protein native states^13−15^ and complexes^16,17^ based only on amino acid sequences. However, the aim of several approaches is not to achieve a reliable (black box) tool for protein structure prediction but to get informative knowledge from the big data available for protein sequences and structures.

Interpretable ML^26,27^ focuses on understanding the cause of a model’s decision and enhancing human capability to consistently predict the model’s result. Interpretable ML versions are more complex and informative than standard statistical analysis and can improve our understanding of proteins.^1,2,4−10^ In particular, they might detect patterns not emerging naturally from studying the abundance and correlations of amino acids in secondary structures. Among the well-known patterns, for instance, there is the amphiphilic structure of several α-helices and β-sheets,^28,29^ which are mostly (charged or) polar  on one side and nonpolar  on the other side. In an α-helix, with pitch of ∼3.6 residues, the typical (non)polarity switch occurs every two residues. On the other hand, in a β-sheet, the three-dimensional alternation of the side chains takes place at every step. Hence, an amphiphilic sequence would be, for example, .

In this work, we use a simple form of interpretable unsupervised ML, restricted Boltzmann machines (RBMs),^30−40^ which allow extraction of deep, nontrivial insight without losing the most transparent information on data statistics encoded in local biases. Conveniently, the weights and biases learned by RBMs can be visualized and easily interpreted. This established approach has already revealed correlated amino acids within protein families,^1^ drug–target interactions,^2^ and correlations within DNA sequences.^41,42^

A novelty of our work is a statistical ensemble approach to unsupervised ML, which improves the robustness of the findings. By training RBMs of the same size but with different weight initializations, we checked whether they all converge to the same final set of learned weights. The maximally complex RBMs preserving this ensemble coherence are optimal, as they perform encoding of the correlations within data samples while providing stable and transparent information on the data.

We show that our optimal RBMs perform extreme information compression to two or three bits, encoding the essential correlations between amino acids at the beginning or end of α-helices and β-sheets. In addition to recovering the expected amphiphilic structures, this approach (i) discovers more subtle yet relevant amino acid patterns in each portion of the secondary structure and (ii) provides evidence of similarity between amino acids’ roles in these structures, including some surprising ones.

RBMs distinguish two classes of amino acids, which we map to  and , as shown in Table 1. Contrary to standard classification,^43^ but similar to some partitionings (see references collected by Stephenson and Freeland^44^), Tyr belongs to the class  of hydrophobic amino acids. Pro is mostly , as discussed in detail below. Some surprising subclasses emerge, especially by looking at the results in α-helices, where it turns out that Thr and His play a similar weak role in the amphiphilic patterns. RBMs classify Asp and Glu on the one side and Val, Leu, Iso, and Phe on the other as the most diverse amino acids. However, Trp has the highest experimental hydrophobicity, while Arg and Lys have the lowest values.^45^ To explain this finding, we argue that RBMs detect a kind of *effective hydrophobicity*, emphasizing how deeply amino acids play a hydrophobic or hydrophilic role in the amphiphilic alternation in α-helices and β-sheets. These findings only partially overlap with those expressed by known diagrams of consensus amino acid similarity.^44^

**Table 1:**
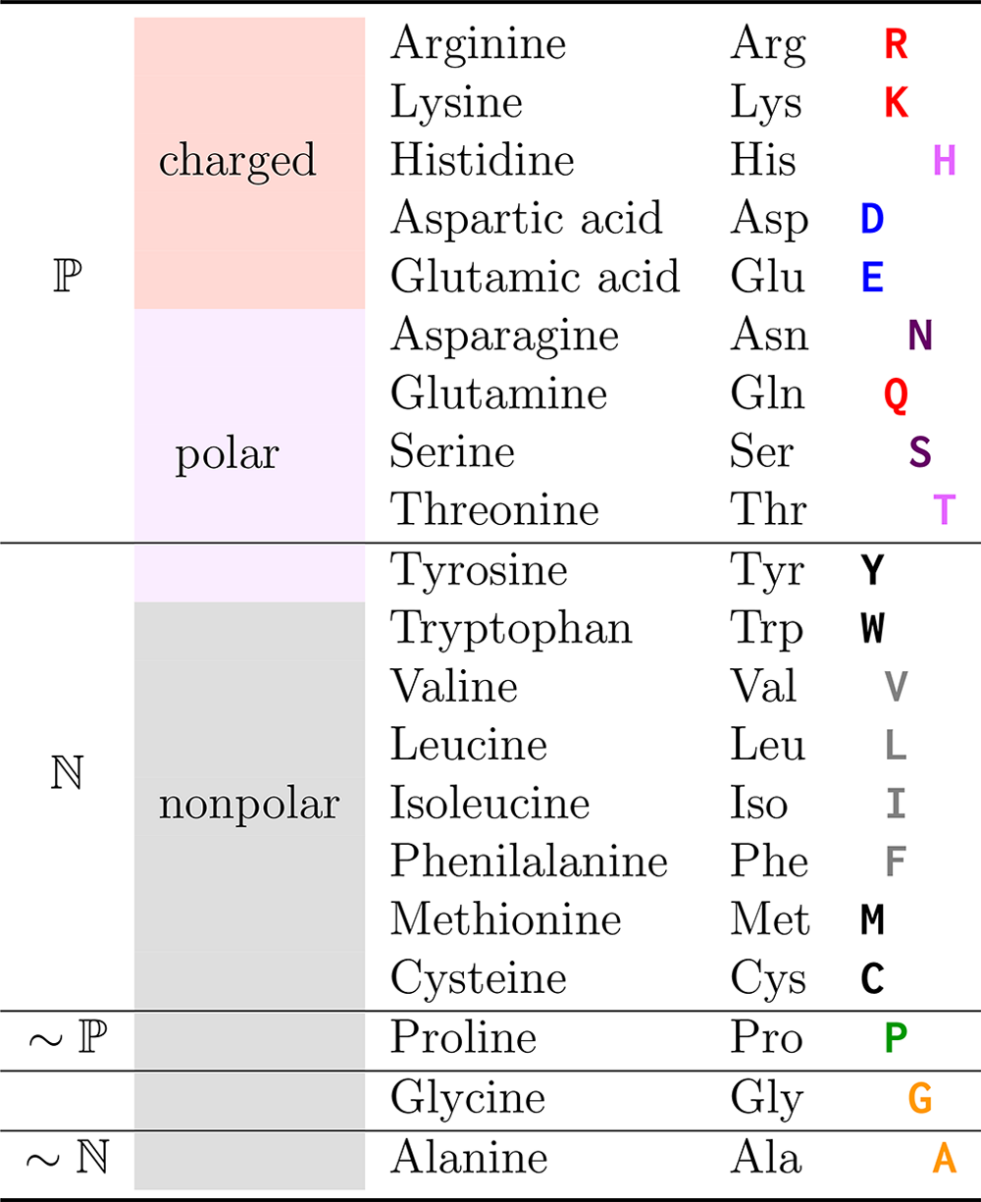
RBM-Based Classification of Amino Acids^a^

a The first column labels which amino acids can be classified as polar/hydrophilic  and nonpolar/hydrophobic  according to the weights of our RBMs. The second column shows the textbook classification of amino acids.^43^ According to RBMs, Tyr behaves as a nonpolar amino acid, Pro behaves mostly as a polar one , and Ala is slightly  only in β-sheets. Gly is neither clearly  nor . The color of each amino acid symbol in the last column (and the offset) follows the subgrouping we introduce based on the weights learned by the RBMs applied to α-helices.

## 2 Methods

Here we describe the datasets and the methods used to analyze them. Some more technical details are reported in Supporting Information (SI) section S1.

### 2.1 Data

To each protein sequence stored in the reduced CATH ensemble of 31 884 natural proteins,^46^ we apply the DSSP algorithm^47,48^ to determine the secondary structure to which every amino acid belongs (see SI section S2 for comparison with results obtained with the STRIDE algorithm^49^). Then we collect all sequences within α-helices and β-sheets long enough to contain Γ = 5 amino acids. We then build four sets: one with the first Γ = 5 amino acids in α-helices (following the standard orientation from the N terminus to the C terminus of the protein), one with the last Γ amino acids in α-helices, and the same for two more sets at the start and end of β-sheets. The two sets referring to the first and last Γ = 5 amino acids in α-helices contain 129300 samples each, while the other two sets, concerning the start and end of β-sheets, include 101 382 sequences each.

We used one-hot encoding to represent amino acids. That is, the *k*th amino acid is stored as a sequence **v**^*k*^ = (−1, −1, ..., +1, ..., −1, −1) of 20 integers where only a +1 element is present at position *k*. This encoding is how an RBM reads the amino acid in a portion of its visible units. A sequence of Γ amino acids (*k*_1_, ..., *k*_Γ_) is thus translated into one-hot encoding stacked as  giving a total of *N*_v_ = 20Γ digits in a data sample.

To monitor the training of each RBM, we randomly split the data into a training set (80%) and a validation set (20%). The training set is used to optimize the RBM parameters and compute the pseudo-log-likelihood (PLL) function, which measures the quality of data reconstruction by RBMs.^50^ The PLL of the validation set is then used to check the performance of the RBM in reproducing the statistics of new data. Note that in principle this procedure could cause differences in the results. However, the cases we find in the ensemble of RBMs, as explained below, reveal when variability is small and highlight general patterns.

### 2.2 Restricted Boltzmann Machines

**Figure 1:**
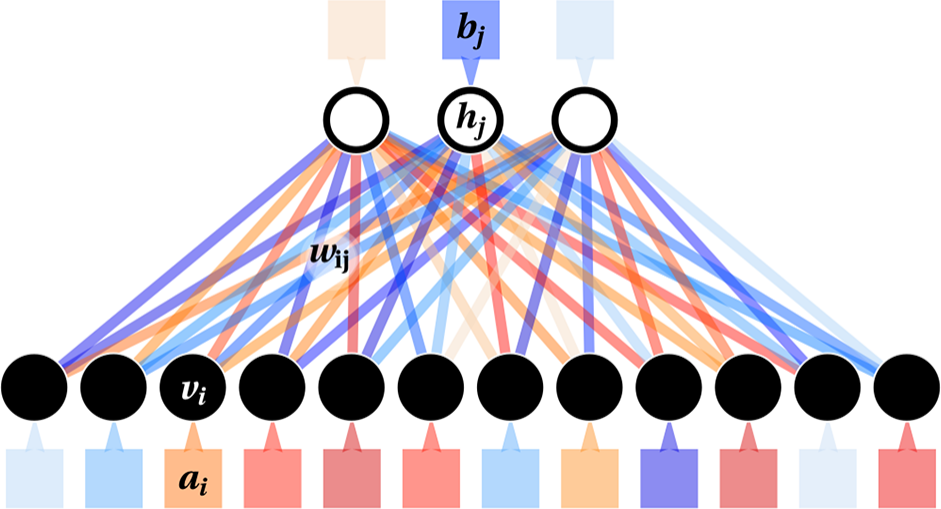
Sketch of an RBM with *N*_v_ = 12 visible units (black circles, where data are given as an input) and *N*_h_ = 3 hidden units (white circles). Red and blue shadings indicate positive and negative values of single weights (plotted as lines joining units in the two layers) and biases (boxes next to units).

The RBM is an unsupervised machine learning method based on a simple neural network architecture. It aims to reproduce the empirical distribution of data samples by encoding the correlations between their elements, the *visible units v*_*i*_ (1 ≤ *i* ≤ *N*_v_). This encoding uses a set of parameters and a layer of hidden (or latent) variables, *h*_*j*_ (1 ≤ *j* ≤ *N*_h_). The parameters defining the method are the weights *w*_*ij*_ in an *N*_v_ × *N*_h_ matrix connecting visible to hidden units and the local biases that act on both the visible (*a*_*i*_) and hidden (*b*_*j*_) units. Figure 1 shows a sketch of an RBM. The statistical weight of a (**v**, **h**) configuration is given by
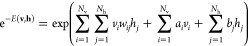
1It resembles a Boltzmann weight with energy *E*(**v**, **h**), for which we will use “spin” variables *v*_*i*_ = ±1, *h*_*j*_ = ±1. Since this version generates a finite number of hidden states (2^*N*_h_^), it facilitates an interpretation of the structure of weights between hidden and visible units and of local biases. Initially, weights and biases of untrained RBMs are drawn randomly from chosen distributions.

The bipartite structure of the RBM allows easy generation of **h** from **v**. This step should encode the correlations within data sequences in *N*_h_ hidden units for a trained RBM. When *N*_h_ ≪ *N*_v_, the RBM acts as an *information bottleneck* enforcing such a simple model, with its small resources, to capture the crucial properties of the analyzed data. The **v** → **h** step selects each *h*_*i*_ independently with probability
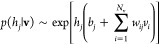
2

Similarly, one generates **v** if **h** is known through **v** ∼ *p*(**v**|**h**). Each of the Γ blocks **v**_γ_ is generated independently. The indices *i* ∈ *I*(γ) of the 20 weights *w*_*ij*_ pointing to segment **v**_γ_ are those relevant for its sampling. By remapping these indices *i* to the interval *k* = 1, ..., 20, we pick an amino acid *k* with probability
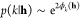
3with
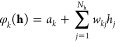
4where φ_*k*_(**h**) is the local field on the site *k*.

The core of the training of an RBM consists of sampling values in visible and hidden units through an algorithm termed contrastive divergence with *n* Monte Carlo steps (CD-*n*) .^51,52^ We alternatively sample from conditional distributions, starting from a data sample **v**_0_ at *t* = 0 up to *t* = *n* steps: **h**_*t*+1_ ∼ *p*(**h**|**v**_*t*_) and **v**_*t*+1_ ∼ *p*(**v**|**h**_*t*+1_). The statistics of the sampled configurations allow the estimation of the gradient of the data log-likelihood according to the Boltzmann weight (eq 1) in the directions of all parameters *w*_*ij*_, *a*_*i*_, and *b*_*j*_. Then we apply a standard gradient ascent algorithm (in our case, Adam^52^). In addition to CD-*n*, we use persistent CD-*n* (PCD-*n*), a variant that should better sample the configurational space.^52,53^

Once trained, the same sampling procedure may generate realistic amino acid sequences (*k*_1_, ..., *k*_Γ_). Through eq 3, an RBM decides how to decode the hidden units **h** and generate a sequence.

### 2.3 Ensemble of RBMs

A novelty of this work is using a statistical ensemble analysis of machine learning. As a trade-off between computational costs and statistical relevance, we run *R* = 30 independent realizations of RBMs with the same size *N*_v_, *N*_h_ and the same number of training epochs (we found that typically 50 or 100 epochs are enough to train the model). Note that each realization differs by the initialization of the weights and the random splitting of the data into training and validation sets.

From now on, let us assume that each hidden unit *j* in a single RBM is characterized by its set of weights *w*_*ij*_ (1 ≤ *i* ≤ *N*_v_). All hidden units from RBM realizations are then collected in an ensemble of hidden units to study their properties and check whether common patterns exist. For every pair of hidden units *j*, *m*, the Euclidean distance  estimates their similarity. When used in a clustering algorithm, it identifies *groups* of hidden units (see SI section S1 for more details).

By averaging the weights *w*_*ij*_ within each group and the biases *a*_*i*_ and *b*_*j*_ of each RBM, we build their average RBM (aRBM): this is supposed to represent the best summary of the relevant information learned by the ensemble. First, we use the aRBM to compute the probability of the 2^*N*_h_^ possible hidden states given that **v** are all points in a dataset (eq 2). Then, from hidden states weighted with their probabilities, we use eq 3 to verify the ability of the aRBM to faithfully reproduce the statistics of the original dataset in the visible space.

### 2.4 Selecting the Number of Hidden Units

The main aim is to find simple patterns representative of the redundant, generic correlations in amino acid sequences (at the start of α-helices, etc.) while neglecting specific patterns of single sequences with RBMs. The key to achieving this goal is the information bottleneck obtained by setting a small number of hidden units *N*_h_.

**Figure 2:**
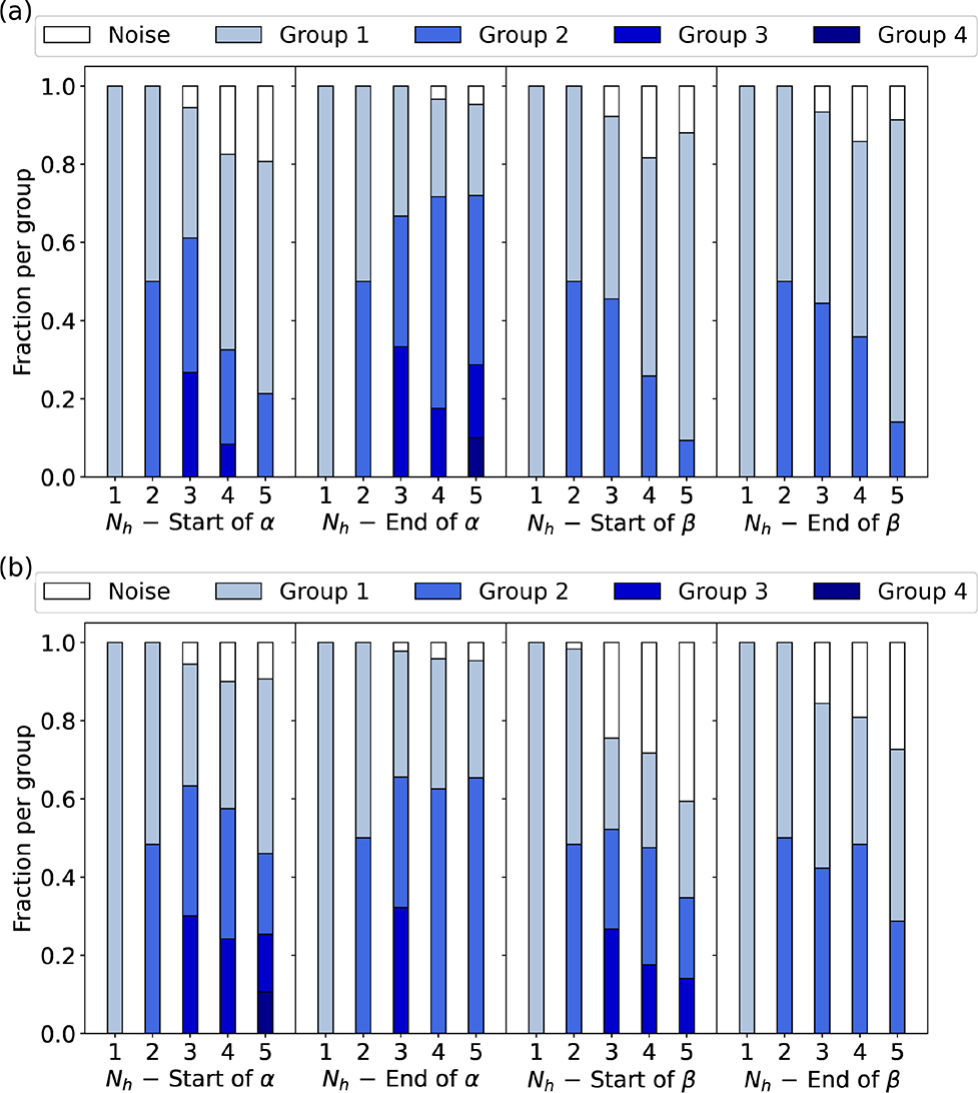
For (a) CD-1 and (b) PCD-10, we show the number and relative size of groups emerging from clustering hidden units in the ensemble of RBMs for every position of the secondary structure that we study. In both cases, we conclude the following: for α-helices, *N*_h_ = 3 is the optimal number of hidden units, while for β-sheets it is *N*_h_ = 2. These are the maximum values where the number of groups matches the number of hidden units and the noise is still tiny, i.e., where each RBM in the ensemble has learned the same set of hidden units.

We monitor how many groups of hidden units emerge by increasing *N*_h_ (Figure 2a for the CD-1 training of RBMs and Figure 2b for PCD-10). Generally, the ratio of groups to hidden units stays maximal up to *N*_h_ = 3 for α-helices and *N*_h_ = 2 for β-sheets. For these values of *N*_h_, almost all RBM realizations have the same palette of hidden units. Only in a few cases does the clustering algorithm (see SI section S1) classify units as *noise* due to their significant diversity from all other ones. Note that beyond these values of *N*_h_, there is no clear one-to-one correspondence between hidden units in an RBM and groups, and uniformity in the ensemble of RBMs is lost.

**Figure 3:**
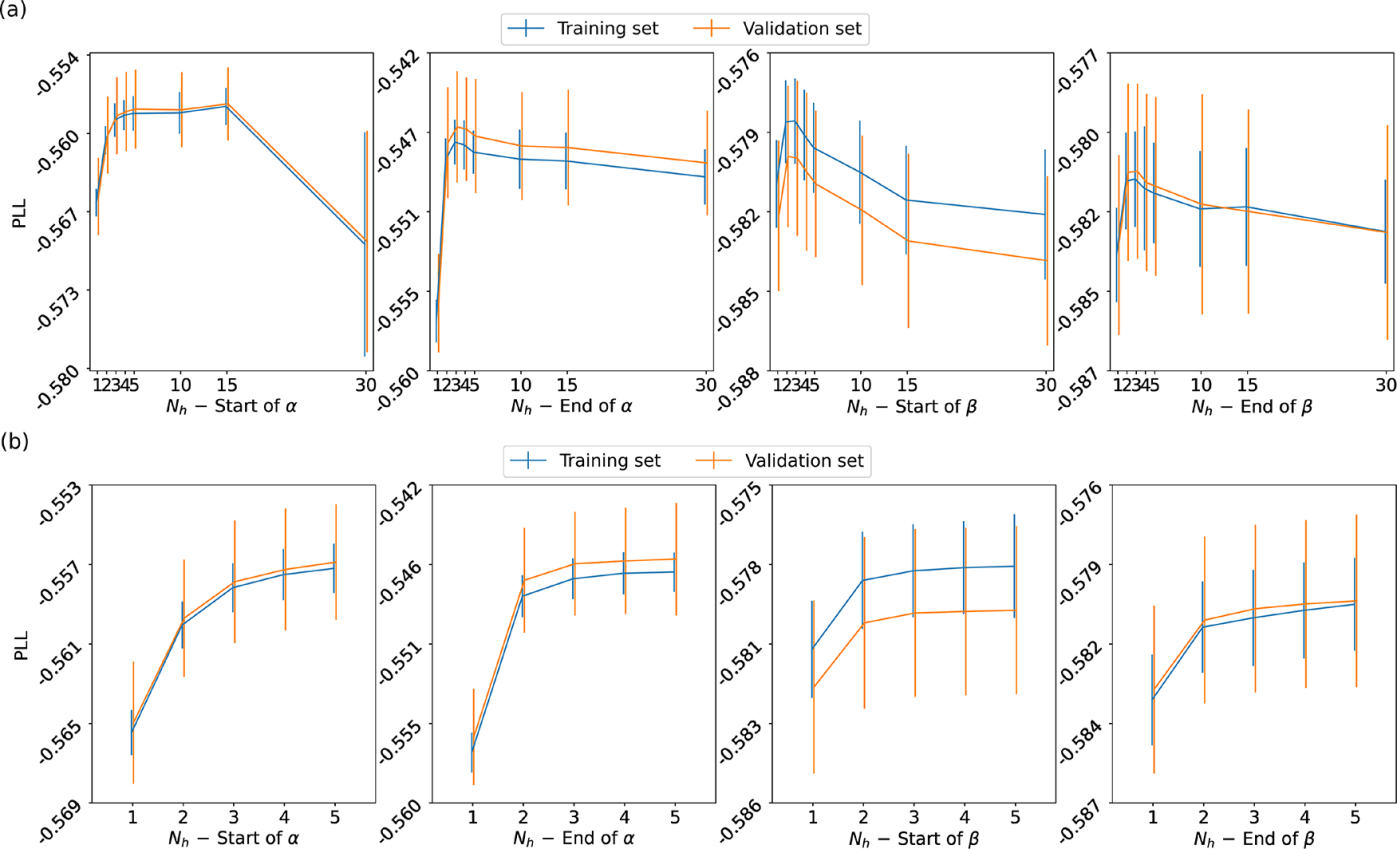
Pseudo-log-likelihood as a function of the number of hidden units for RBMs trained with (a) CD-1 and (b) PCD-10, shown for each of the four segments of secondary structure that we study. The PLLs for the training and validation sets are compatible, showing that the RBMs have achieved robust training.

To evaluate the performance of the RBMs, we compute the PLL as a function of *N*_h_ (see Figure 3). From *N*_h_ = 1, for CD-1 and PCD-10, the PLL quickly reaches a plateau around *N*_h_ ≈ 3. By adding more hidden units (*N*_h_ > 3), one does not significantly improve the performance of the RBM. Moreover, for CD-1 we can go up to *N*_h_ = 30, finding a decreasing trend of the PLL for large *N*_h_ values in all cases. Hence, more complex RBMs are heterogeneous and suboptimally trained by the oversimplified CD-1 algorithm.

All considered, we show the results for *N*_h_ = 3 for α-helices and *N*_h_ = 2 for β-sheets. From now on, we will discuss only the results from PCD-10. Those from CD-1 are similar.

## 3 Results

### 3.1 How to Read Weight Patterns

**Figure 4:**
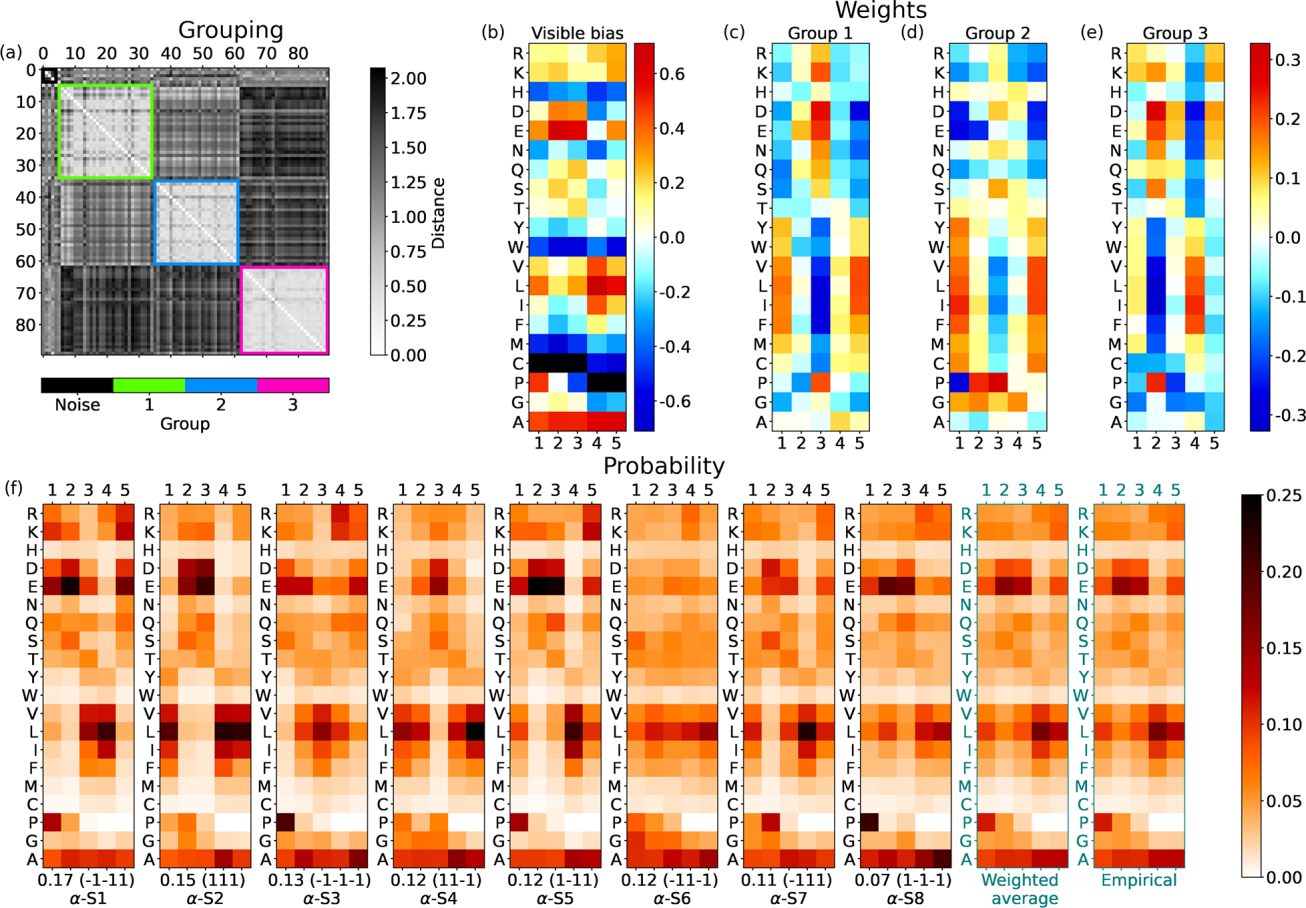
For the start of α-helices, with *N*_h_ = 3 hidden units: (a) Matrix with gray shade indicating the distance *d*_*jm*_ between the weights *w*_*ij*_ and *w*_*im*_ of different hidden units *j* and *m*; the colored boxes highlight the groups found by the DBSCAN clustering. (b) Average biases *a*_*i*_ learned by the ensemble of RBMs, reshaped from an array with 20Γ = 100 entries to a 20 × Γ table, in which each column corresponds to a given encoding **v**_γ_ and each row to a given amino acid (a similar scheme is used in (c–e)). Values more negative than the lower threshold in the scale are marked with black squares (in this case for Cys and Pro, which essentially leads to the negligible probability of finding these amino acids in those positions). (c–e) Average weights of units in groups 1, 2, and 3. (f) The shade of each slot in each panel shows the probability of picking a specific amino acid at a given position. Hence columns are normalized to 1. The first 2^*N*_h_^ = 8 panels show the probabilities for every hidden state (the sequence of ±1’s in the parentheses at the bottom, where it follows the value of its empirical frequency). Hidden states are labeled and ranked with decreasing frequency; e.g., α-S1 is the most probable hidden state at the start of α-helices. The last two panels show the average of RBM α-S states weighted according to their frequency and the actual probability of amino acids at the Γ = 5 initial positions of α-helices. In practice, the prescription of the RBM for reconstructing meaningful sequences would be (i) to pick a hidden state at random according to its frequency and (ii) according to probabilities in its table, for every position γ ≤ Γ, to pick an amino acid at random. The values of the hidden bias in the aRBM for each group are *b*_1_ = −1.129, *b*_2_ = 1.270, and *b*_3_ = −1.496.

We summarize the properties of the ensemble of RBMs via a set of plots, as reported, for instance, in Figure 4 for the starting strand of α-helices. We average the values for weights in a group or biases from all RMBs in the ensemble. Thus, the displayed values represent the aRBM.

Figure 4a reports the table of distances between any two hidden units of the ensemble of RBMs. The units are sorted and collected into the groups (colored squares with light internal colors along the diagonal) detected by the clustering algorithm. Some units, marked as “noise”, are not assigned to any group.

Figure 4b shows the visible bias *a*_*i*_ of the aRBM, reshaped to a 20 × Γ matrix for better readability. The exponential of this bias is a good indicator of the mean probability of finding an amino acid at a specific position in the sequence. For instance, it shows that Glu (E) has a high chance of appearing at positions 2 and 3. For each of the groups, we show in Figure 4c–e the weights *w*_*ij*_ of the corresponding hidden units in the aRBM. In addition, the *N*_h_ biases *b*_*j*_ of the aRBM are specified in the caption.

A table such as the one reported in Figure 4c may be read as follows: a hidden unit in group 1 “pushes” a random pattern of amino acids biased by the weights toward a particular sequence, depending on its stored value, *h*_1_ = ±1. According to eqs 3 and 4, *h*_1_ = 1 raises the probability of picking amino acids with weights *w*_*i*1_ to a value significantly larger than zero (red shades). For instance, one can notice that D is chosen more frequently at position 3 and I, L, V, and F at positions 1 and 5. The opposite happens if *h*_1_ = −1.

All other random choices are possible with a gradually lower probability. The unit does not (de)select any particular amino acid when weights have values close to zero (light colors in the table). Instead, another unit may be the one that drives the sequence selection at that position. For instance, in Figure 4c,d we see that units in group 1 and group 2 have a strong set of weights at positions 1, 3, and 5, which are complementary to those of group 3 (stronger at positions 2 and 4; see Figure 4e). Therefore, the units in different groups may take care of different alternating slots in the sequence.

The first 2^*N*_h_^ = 8 panels in Figure 4f represent probabilities (eq 3) to choose amino acids at every slot γ ≤ Γ (normalized in columns at fixed γ) if the aRBM is in a given state **h**. With eq 2, we compute the probability of each of the 2^*N*_h_^ = 8 hidden states **h** from the biases *b*_*j*_, weights *w*_*ij*_, and **v** in the dataset. This is indicated below each panel in Figure 4f (e.g., 0.18). After this, we also specify the state **h** (e.g., (1 −1 −1)) and a chosen label (e.g., α-S1). We rank the **h** states in Figure 4f from the most frequent to the least likely.

Each of the first 2^*N*_h_^ panels of Figure 4f thus displays a typical correlation of probabilities followed by the aRBM to build a sequence of amino acids. The last two panels are the average of the first 2^*N*_h_^ panels, weighted with their frequencies, and the empirical average in the dataset.

In the following, we specify the discussion of the four regions of the secondary structure analyzed in this work.

### 3.2 Start of α-Helices

The first training set we study contains stretches of the first Γ = 5 positions in all (long enough) α-helices in proteins of the CATH database. The corresponding set of trained RBMs with *N*_h_ = 3 yields three significant groups of hidden units (see Figure 4a). For *N*_h_ = 1, 2, 3, we see groups 1, 3, and finally 2, respectively. By including additional hidden units, we continue to observe these three groups, confirming that RBMs encode the main patterns within the analyzed sequences with three hidden units.

Figure 4b shows the bias *a*_*i*_ of the aRBM. It is quite structured compared to other cases, shown later as the end of α-helices and β-sheets. This structure denotes a tendency of amino acids to appear more frequently at specific positions. Notice the pattern of Pro, with high intensity (red) at the first position, which sensibly decreases in the next positions (the black color means that *a*_*i*_ is below the lower level of the scale), in agreement with the known abundance of Pro at the start of helices.^54^ Notably, at position γ = 4, there stands out a peculiar behavior: a high intensity for nonpolar amino acids (in particular Val (V), Leu (L), and Iso (I)) aligns with a low intensity for polar amino acids (especially Asp (D), Glu (E), and Asn (N)). Consistently, an average depletion of a polar amino acid at position γ = 4 at the start of α-helices is visible in the empirical statistics, shown in the last panel of Figure 4f.

In addition to the average trend dictated by the bias, the aRBM, thanks to the hidden units, can modulate the correlations among amino acids in single sequences. Hidden units in group 1 (Figure 4c) address anticorrelations between  and  amino acids at positions γ = 1, 3, 5. For instance, *h*_1_ = 1 promotes the pattern -- while *h*_1_ = −1 promotes --. Group 3 (Figure 4e) instead mainly encodes the correlations among amino acids at positions γ = 2, 4. Group 2 (Figure 4d) is similar to group 1 but also displays a set of large weights for Pro. This set adds significant insight into the correlations between Pro as a starter of helices and its following amino acids (the bias did not show such a rich structure): for example, weights in group 2 suggest that P, E, and D are interchangeable at the position γ = 1 and that they are strongly correlated with D and E at γ = 5 and anticorrelated with P at γ = 3.

Given the aRBM, we check the states in the hidden space in Figure 4f, allowing us to merge the information from biases and weights. Different configurations appear, but almost all show a repeated scheme with polar and nonpolar amino acid alternation with blocks of about two elements, consistent with an amphiphilic structure in α-helices. More interestingly, states α-S1, α-S3, α-S5, and α-S8 (sharing *h*_2_ = −1 that promotes Pro in group 2) include the activation of Pro at the start of the sequence, paired with Glu in the second position (for this subset of α-helices, we notice that Glu’s activation is not fixed only at the second position but is active also at the first or third position). This pattern provides two main classes of amino acid alternation: (Pro) or (Pro). In this context, Pro behaves as polar, with a higher frequency (α-S1, α-S3), or as nonpolar, with a lower frequency (α-S5, α-S8).

We have thus shown that training led the RBMs to automatically detect and decompose the start of α-helices into eight nontrivial modes. The reverse, trivial process of averaging their probabilities leads to the average behavior shown in the second-to-last panel of Figure 4f, which matches the empirical probabilities (last panel). Notably, the RBM decomposition would not be accessible a priori by standard statistical tools. Moreover, the discovered heterogeneous eight modes *generate* synthetic sequences, each with its own probabilistic pattern.

### 3.3 End of α-Helices

**Figure 5:**
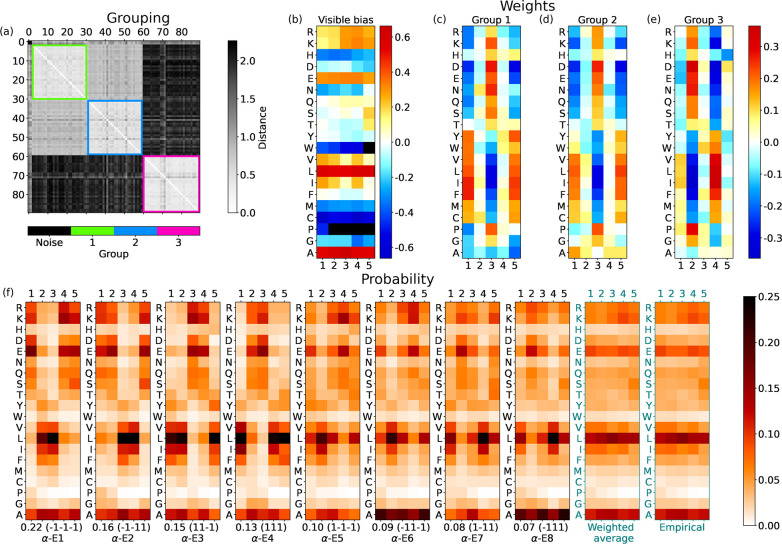
For the end of α-helices with three hidden units, the same scheme as in Figure 4. Hidden bias: *b*_1_ = 0.541, *b*_2_ = −0.410, and *b*_3_ = −0.191.

The results from RBMs with *N*_h_ = 3 for the last Γ = 5 amino acids of the α-helices are displayed in Figure 5. Again, three groups of hidden units emerge from clustering their weights. For *N*_h_ = 4, the values would remain the same. However, by increasing *N*_h_ from *N*_h_ = 1, we note that groups 1 and 2 are represented by their averaged version for *N*_h_ ≤ 2, while they split for *N*_h_ = 3. This splitting is convincing: indeed, the PLL slightly increases in the *N*_h_ = 2 → 3 step (Figure 3), and above all, the division into separate groups by the clustering is clear (see Figure 5a).

Groups 1 and 2 determine the alternation of  and  at positions γ = 1, 3, 5. What distinguishes them is the weight pattern of Ala, which flips its sign from one group to the other (see Figure 5c,d. Group 3 instead fixes the alternation of  and  at positions γ = 2, 4.

The visible bias in Figure 5b shows that amino acids distribute almost uniformly at different positions at the end of α-helices, with a significantly high bias toward Leu (L) and Ala (A). However, some slight deviations from the general behavior are visible. For example, at the last position of the helix (γ = 5), some polar amino acids are more probable (see N, S, and T), while some nonpolar ones become less likely (see V, I). Note also the low bias of Gly at the next-to-last position (γ = 4).

In the hidden space, the aRBM reproduces, on average, the visible statistics (see Figure 5f). As observed at the start of α-helices, some states (α-E1, α-E2, α-E3, and α-E4) report the polar and nonpolar alternation with period ∼2. More interestingly, in states α-E6 and α-E8, the behavior of Ala spikes with a high probability in every position. As known, Ala is a strong helix stabilizer.^55,56^ Consistently, Ala has a high bias *a*_*i*_ in the RBM and thus can act as a wild card: its placement in a typical sequence at the end of an α-helix is relatively free, and it fits even at the specific positions of charged and polar amino acids. This high bias was also visible at the start of α-helices (Figure 4b), where no weight pattern induces the splitting of hidden units into separate groups based on Ala. The boosted probability of Ala in states α-E6 and α-E8 reveals a subclass of α-helix endings (15% of the cases) richer in Ala than typical α-helices. We have verified a posteriori that AAAAA is among the 10 most frequent sequences at the end of α-helices. Hence, polyalanine^56^ is a characterizing feature of the terminal part of α-helices.

Finally, note that the patterns of the states α-E1, α-E2, α-E3, and α-E4 are similar but somehow shifted. Our explanation is that α-helices may end with different “phases” for exposure to the solvent. In some cases, it is convenient for the last amino acids of a helix to be polar; in others, it is the opposite. Patterns in α-E5, α-E6, α-E7, and α-E8 show shifts of polarity that satisfy different needs.

### 3.4 Start of β-Sheets

An alternating sequence  of polar and nonpolar amino acids may allow β-sheets to expose side chains of the same kind at each of their two sides, making them amphiphilic. For *N*_h_ = 1, we find that the single hidden unit has weights of alternating signs with γ and opposite polarity for  and , which would often lead to generating amphiphilic sequences. However, not all β-sheet stretches follow this simple amphiphilic scheme. For *N*_h_ = 2, two groups emerge from clustering. The three hidden unit groups emerging for *N*_h_ = 3 instead invalidate the analysis based on the aRBM for two reasons. First, many units are considered noise by the clustering algorithm; second, within single RBMs, we find high heterogeneity in the combination of groups. Therefore, we choose *N*_h_ = 2 as the optimal number of hidden units leading to the most consistent yet complex aRBM. In support of this choice, note that the most significant increase in the PLL occurs from *N*_h_ = 1 to *N*_h_ = 2 (Figure 3).

**Figure 6:**
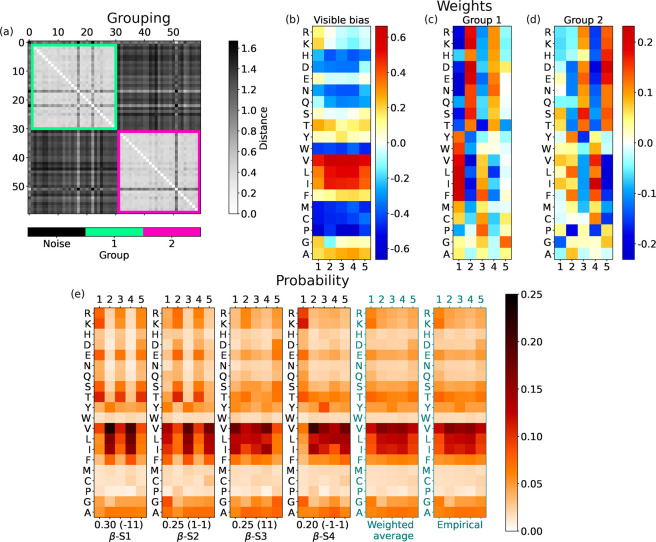
For the start of β-sheets with two hidden units, the same scheme as in Figure 4. Hidden bias: *b*_1_ = 0.458 and *b*_2_ = −0.002.

The weights of the two groups preserve the  alternation only at the beginning (group 1, Figure 6c) or at the end (group 2, Figure 6d). These will yield a hidden state **h** compatible with the amphiphilic pattern of weights if combined with the proper signs of *h*_1_ and *h*_2_: the probability of amino acids for mode β-S1 (Figure 6e) promotes the  alternation, while that for mode β-S2 promotes the  pattern. They cover 55% of the cases.

However, the remaining 45% of combinations of hidden states suppress the  alternation, and β segments  (mode β-S3) and  (β-S4) are more likely to be generated by RBMs. In particular, β-S4 shows a strong activation of polar amino acids in the first position of β-sheets in comparison to the aliphatic ones, which are instead very favored in the next four positions.

The weighted average of probabilities for β-S1···4, as before for α-helices, matches the empirical distributions (last panels of Figure 6e). The RBM-learned decomposition thus splits the start of β-sheets into four modes: the first two modes promote amphiphilic patterns, and the last two modes favor uniform stretches of four ’s (mostly I, L, V) capped by a different type of amino acid. This decomposition somewhat joins previous results in which the amphiphilic alternation of β-sheets was seen by different works, with more straightforward statistical tools, either over-represented^57^ or under-represented.^58^

The bias *a*_*i*_ at the start of the β-sheets shows a uniform distribution of amino acids at different positions of the chain (see Figure 6b). For instance, aliphatic amino acids show a high bias. However, for a small subset of amino acids, there emerges variability. For example, Arg (R) and Lys (K) have a decreasing bias from the first position in the β strand to the following ones. Perhaps the most interesting behavior is observed for Gly, with a high bias except at the second position (γ = 2), suggesting that Gly is not likely to appear there.

### 3.5 End of β-Sheets

**Figure 7:**
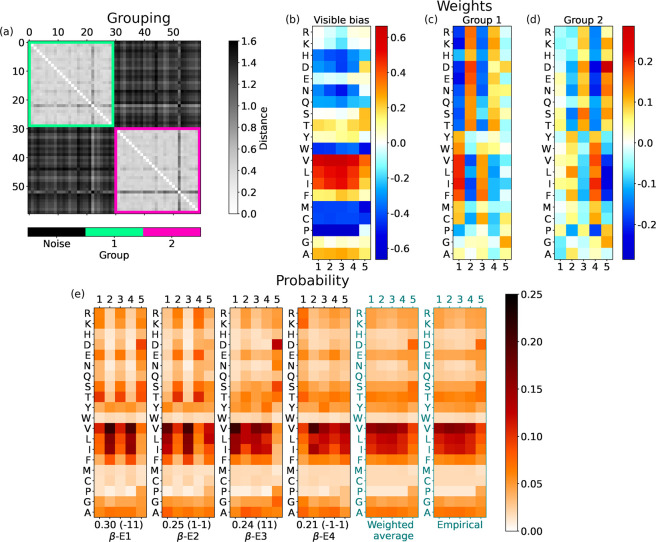
For the end of β-sheets with two hidden units, the same scheme as in Figure 4. Hidden bias: *b*_1_ = −0.349 and *b*_2_ = −0.226.

Generally, the analysis of the end of β-sheets retraces the start of β-sheets. Thus, on average, the ensemble of RBMs can capture only patterns of little complexity in β-sheets compared with those of α-helices. We take *N*_h_ = 2 also for the end of β-sheets, and again we observe two groups similar to those at the start of β-sheets (Figure 7c,d).

Visible biases (Figure 7b) show a uniform distribution of amino acids at different positions close to the ends of β-sheets. However, there is a significant increase in the bias at the last position (γ = 5) for many small amino acids. Furthermore, many of these are polar (Asp (D), Asn (N), Ser (S), Thr (T)), and there is also Pro (P). In the next section, we will stress that Pro is often positively correlated with polar amino acids. The biases shown in Figure 7b are different from those found at the start of β-sheets (Figure 6b). As a consequence, the probabilities in Figure 7e diverge slightly from those in Figure 6e. In particular, mode β-E3 promotes sequences such as , (Pro), or (Gly).

One may notice some similarity between modes β-S in Figure 6e and modes β-E in Figure 7e. This is due to an overlap of 20% of sequences between the two datasets corresponding to β-sheets with five residues. This is not seen for α-helices, which are on average longer, with only 3% having a length of five residues.

### 3.6 Amino Acid Similarities

The abundance or absence of a given amino acid in α-helices or β-sheets is primarily encoded in the visible biases *a*_*i*_. One can check that they correlate with results from standard statistical analysis.^59^ However, these biases are not directly related to the polarity or size of amino acids. Hence, they do not provide complete information about the amino acid patterns in secondary structures.

The refined information on amino acid similarities is given by the weights shown in panels (c), (d), and eventually (e) of Figures 4–7. Each row in a panel shows the weights of a given amino acid in that group. The similarity of amino acids in a group emerges when their weights are interchangeable; i.e., the Γ = 5 weights appearing in the row of a given amino acid can be swapped with the other ones in a row of an equivalent amino acid without a significant change in the whole set of weights *w*_*ij*_ of the corresponding hidden unit *j*.

**Figure 8:**
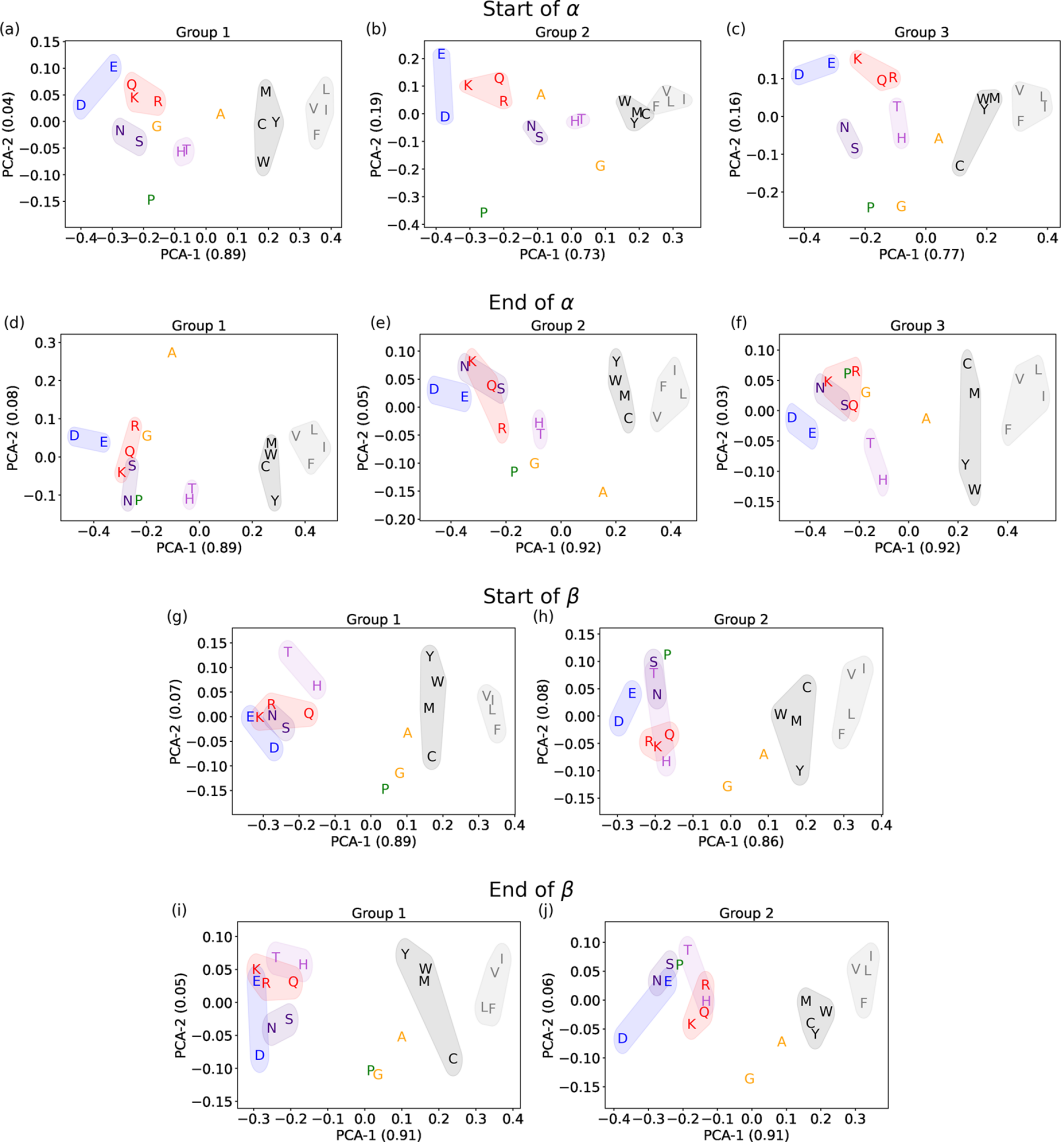
Principal component analysis of amino acid weights. Each panel shows the first two components of the PCA for each amino acid in a hidden-unit group for a given part of the secondary structure. The numbers in the axis labels in the parentheses are the average explained variances. Color-shaded ensembles and single amino acids are discussed in the text. (a–c) Groups at the start of α-helices, shown in Figure 4. (d–f) Groups at the end of α-helices (Figure 5). (g, h) Groups at the start of β-sheets (Figure 6). (i, j) Groups at the end of β-sheets (Figure 7).

In our unsupervised machine learning approach, the salient traits of amino acids’ similarity emerge from principal component analysis (PCA). For each hidden unit, we compute the PCA of the Γ = 5 weights associated with each amino acid. Then, for all groups shown in Figures 4–7, we show these two PCA components in Figure 8 to check amino acid similarities. The number in each axis label represents the average variance *explained* by each PCA component, measuring its relevance. In all cases, the first component of PCA, PCA-1, explains the major part of the variance and is related to the polarity of the amino acids.

Families of interchangeable amino acids emerge, as highlighted in all panels of Figure 8. Let us discuss our interpretation of these plots by collecting similar amino acids in small coherent groups. We define this by looking primarily at their PCA components in α-helices, where there is a clearer subdivision. Our amino acid cataloging was anticipated in Table 1.

#### Aspartic Acid (D) and Glutamic Acid (E)

These negatively charged amino acids are always at the left boundary of the PCA-1 component. Looking at the general arrangement of amino acids in the panels of Figure 8, we interpret this as a signal of a strong hydrophilic tendency that stands out even among charged and polar amino acids. Indeed, in all cases, the Pearson coefficient between PCA-1 and the hydrophobicity is ∼0.9. Although Asp and Glu seem mostly similar, for the end of β-sheets, in Figure 8j, we observe that Asp (D) stands away from other polar amino acids. This indicates that Asp has a special role at the end of the β-sheets, which cannot be implemented even by Glu.

#### Asparagine (N) and Serine (S)

The PCA always shows these two small polar amino acids very close to each other. Moreover, they are placed between the pair of Asp and Glu and the central part of the PCA-1 component. This should be related to their lower hydrophilic tendency.

#### Lysine (K), Arginine (R), and Glutamine (Q)

These amino acids have positively charged (K, R) or polar (Q) long side chains. They appear similar, and for them, we can retrace the comments just made for Asn and Ser.

#### Histidine (H) and Threonine (T)

Histidine is a weakly positively charged, large (>150 Da) amino acid, while Thr is a polar, small (<120 Da) amino acid. Thus, it is surprising to find them very well paired in the PCA plots for α-helices, where they sit in a middle region and are not very close to those of other hydrophilic amino acids. Hence, His and Thr display a similar weak tendency to contribute to the amphiphilic pattern in α-helices. In β-sheets, instead, they are not so correlated and are more overlapped with other polar amino acids.

#### Tyrosine (Y), Tryptophan (W), Methionine (M), and Cysteine (C)

These amino acids always have very similar PCA values on the right side of the panels. This quartet comprises a duo of aromatic amino acids (Y, W) and a duo of nonpolar amino acids with sulfur (M, C). In particular, Cys is a small, unique amino acid that can form disulfide bonds. Yet, the PCA correctly places it in the mild hydrophobic region (i.e., with positive but not extreme PCA-1 values).

#### Valine (V), Leucine (L), Isoleucine (I), and Phenylalanine (F)

Three aliphatic amino acids (V, L, and I) and Phe are always equivalently set on the rightmost side of the PCA-1 component. Our analysis with RBMs thus reveals that these four amino acids should be regarded as the strongest hydrophobic amino acids.

#### Alanine (A)

Ala shows neither a clear hydrophobic tendency nor a clear hydrophilic tendency in the PCA plots of α-helices (Figure 8a–f). Nevertheless, we find a peculiar isolation of Ala from the other amino acids in groups 1 and 2 of the end of α-helices (Figure 8d,e), with an opposite sign of the PCA-2 component in the two cases. As discussed above, this is related to the unique role of Ala in helices, in particular at their end, where stretches of five Ala are not rare. However, in β-sheets, Ala shows a mild tendency to cluster with the  group and thus behave as hydrophobic (Figure 8g–j).

#### Glycine (G)

Even if Gly is a nonpolar amino acid, in α-helices it is mainly found in the region populated by hydrophilic amino acids. However, this is not the case in β-sheets, where Gly is not affiliated with other groups.

#### Proline (P)

Similarly to Gly, Pro is not polar but is often aligned with polar amino acids along PCA-1. However, P displays several extreme values of PCA-2, which isolate it from the other amino acids. The most striking case is in group 2 at the start of α-helices (Figure 8b), which RBMs use to highlight the importance of Pro in this portion of the secondary structure.

Before concluding, we note that our PCA plots are similar to the embeddings learned by much more complex neural networks using Transformers.^12^ That analysis showed that the machine catalogs amino acids based on their biological properties.

## 4 Conclusions

We introduce and showcase how an ensemble analysis of (unsupervised) machine learning models, based on restricted Boltzmann machines (RBMs) and with an information bottleneck in encoding data correlations, offers a relatively easy reading of precise yet unexpected similarities between amino acids and emphasizes essential features for building secondary structures. Besides recovering a way to promote the frequent amphiphilic design of α-helices and β-sheets, RBMs discover that there are relevant motifs that, to the best of our knowledge, are not known.

The most diverse scenario is at the start of α-helices. RBMs recover the known relative abundance of Pro in their first positions and promote it to the role of a highly relevant feature in addition to amphiphilicity. Moreover, RBMs add information on correlations between Pro and other amino acids, particularly Asp and Glu, which lead to two typical types of helices starting with Pro. Our complete analysis reveals a frequent alignment of Pro with polar amino acids.

At the end of α-helices, there emerges a particular behavior of Ala, which is the distinguishing amino acid between two otherwise similar amphiphilic patterns. This bimodality implies that in nature there is a class of α-helices closed by stretches richer in Ala than in typical helices.

Moreover, our analysis allows refining of the separation between polar and nonpolar amino acids, highlighting intriguing subclasses. The most unexpected is the coupling of His and Thr in α-helices, where they do not contribute to the amphiphilic patterns. Then, for instance, we found the coupling of Phe with the aliphatic amino acids or the alignment of Trp with Tyr, Met, and Cys.

The first component of our PCA (PCA-1) is strongly correlated but does not precisely follow the hydrophobicity ranking reported in the literature. Nevertheless, PCA-1 explains most of the fluctuations of weights in the RBM. Hence, it is crucial to unveil its meaning. We conjecture that PCA-1, the main feature learned by RBMs to reproduce realistic alternations of polarity in secondary structures, expresses a form of *effective hydrophobicity*. In other words, it reveals how much an amino acid, in α-helices and β-sheets, is mainly focused on the role of being either hydrophobic or hydrophilic. For example, even if it is not the most hydrophilic amino acid, Asp most often displays the strongest negative PCA-1 value (and has a special role in closing β-sheets).

One may wonder whether results similar to ours could emerge from a standard approach based, for instance, on two-site correlations from the sequence data, which requires the computation and parallel visualization of many matrices (see SI section S3). This procedure makes recognizing and interpreting some meaningful patterns possible but far from being naturally summarized in a simple set of ranked-by-relevance multisite correlations, as achieved by an analysis of RBM weights with an increasing number of hidden units. Note, moreover, that RBMs can generate sequences, which is not possible with correlation matrices.

To conclude, the RBM is a simple unsupervised machine learning method that retrieves known results and enriches previous knowledge. Moreover, the RBM’s architecture is readable and, with some effort, interpretable, yielding nontrivial information that is inaccessible by standard statistical tools. For example, we have provided an interpretation of the RBM weights in our study of amino acid patterns and similarities in secondary structures. However, the richness of the results may allow the reader to notice additional details of the arrangement of amino acids in the secondary structures.

## Data Availability

The code used in this work is available upon reasonable request.

## Supporting Information Available

Details on the implementation and training of RBMs and on the clustering of hidden units (section S1), comparison between DSSP and STRIDE algorithms for α-helices (section S2), and matrices of correlations between amino acids (section S3) (PDF)
