# Supplementary material for: Interpretable Machine Learning of Amino Acid Patterns in Proteins: A Statistical Ensemble Approach
Source: J Chem Theory Comput. 2023 Aug 8;19(17):6011–22. doi: 10.1021/acs.jctc.3c00383 (PMC10500975; doi:10.1021/acs.jctc.3c00383)
Supplement: Supplementary file 1 — ct3c00383_si_001.pdf [file ct3c00383_si_001.pdf]

Supplementary Information

Interpretable machine learning of amino acid patterns in proteins: a statistical ensemble approach

Anna Braghetto,[†,‡] Enzo Orlandini,[†,‡] and Marco Baiesi[*,†,‡]

†*Department of Physics and Astronomy, University of Padova, Via Marzolo 8, Padua, Italy*
‡*INFN, Sezione di Padova, Via Marzolo 8, Padua, Italy*

E-mail: marco.baiesi@unipd.it

S1 Details on the algorithms

We provide additional details on the restricted Boltzmann machines (RBMs) and on the clustering procedure.

Our RBMs are trained with well-known optimizations[1,2] and with the "centering trick".[3,4] We build the ensemble of RBMs, with a fixed number of hidden units N_h, by training R different realizations. Each realization is characterized by the RBM's random state, which determines the weights initialization and the data split into training (80%) and validation (20%) sets. Thus, we obtain a set of RBMs differing for parameter values and slightly for analyzed datasets. Each weight w_{ij} is initially drawn from a uniform distribution in the interval $[-\ell, +\ell]$ with $\ell = 2(N_h + N_v)^{-1/2}$. Biases are initialized to zero.

The bipartite structure of the RBM allows storing the information within weights and biases in many ways due to the invariance for permutation and sign reversal of hidden units. To overcome this variability when comparing units, we isolate each hidden unit j in an RBM and compare its weights w_{ij}, and those of its mirror image $-w_{ij}$, with those of all other hidden units in the same RBM and other RBMs of the ensemble. Thus, for every pair of hidden units j, m, we compute the minimum Euclidean distance among them or their mirror versions,

$$d_{jm} = \min \left[\sqrt{\sum_i |w_{ij} - w_{im}|^2}, \sqrt{\sum_i |w_{ij} + w_{im}|^2} \right] . \tag{S1}$$

We then feed the distance matrix d_{jm} to a popular density-based algorithm, DBSCAN,[5,6] to perform clustering. We focus on tuning two parameters: a radius around each data point (ϵ) and the minimum number of samples (min_s) within ϵ from a data point that would prevent its labeling as *noise*, i.e., that would grant to put that point in a cluster.

For tuning ϵ and min_s, we introduce a cost function $C(\epsilon, min_s)$ whose minimum corre-

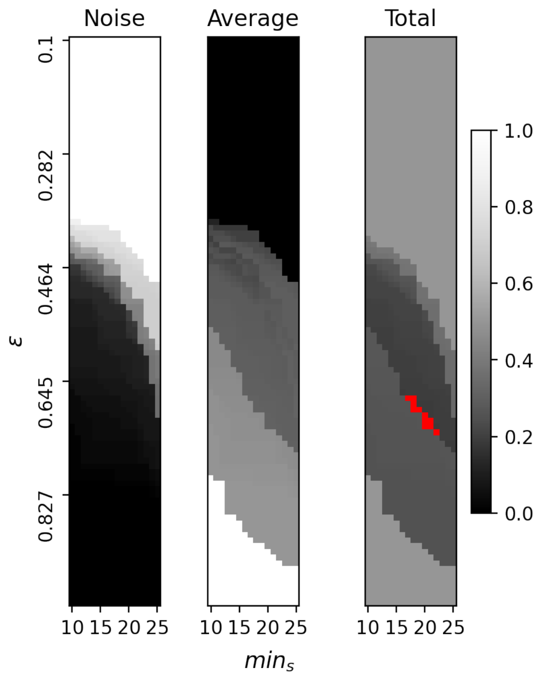

Figure S1: Example of parameter tuning in DBSCAN for the start of α-helices. The "Noise" matrix represents the fraction of noise points (first term in (S2)). The "Average" matrix represents the average group size divided by the total number of hidden units in the ensemble (second term in (S2)). The "Total" matrix is the sum of the previous two and coincides with the cost function in (S2). The red points highlight the optimal region of the parameters.

sponds to the optimal parameter values,

$$C(\epsilon, min_s) = \left[1 - \sum_{g\in\mathcal{G}} \frac{\Omega(g)}{R\,N_h}\right] + \left[\frac{\langle\Omega(g)\rangle_{g\in\mathcal{G}}}{R\,N_h}\right] \tag{S2}$$

where $\mathcal{G} := \mathcal{G}(\epsilon, min_s)$ denotes the set of groups returned by DBSCAN and $\Omega(g)$ denotes the number of hidden units in group $g \in \mathcal{G}$. The first term in (S2) is the fraction of hidden units that are cataloged as noise. Hence it penalizes configurations with high noise. The second term is proportional to the average cluster size $\langle\Omega(g)\rangle_{g\in\mathcal{G}}$ and penalizes configurations with all the hidden units merged in a single, giant cluster. In Figure S1 we show the results and the intermediate steps of the parameter tuning at the start of α-helices. For the other cases, the results are similar. The procedure returns an optimal region of parameters: we choose the average points within this region as optimal parameters. In Table 1, we collect the optimal hyperparameters of DBSCAN analysis for each dataset.

Table 1: Optimal hyperparameters of DBSCAN analysis.

Structure	ϵ	min_s
Start of α-helices	0.69	19
End of α-helices	0.56	17
Start of β-sheets	0.76	19
End of β-sheets	0.72	17

The average RBM is then built as the average of weights w_{ij} and biases b_j within each group after aligning all its hidden units to minimize the distance from a reference one. For

better overall visualization, since aliphatic amino acids (I, L, V) always yield a well-defined pattern, we adopt the convention that their weights at position $\gamma = 1$ are positive. The visible bias a_i is instead independent of the grouping; hence, it is averaged among all the original RBMs.

For each hidden unit j representing the average of units in a group, we analyze weights w_{ij} to extract the similarity among amino acids. For this purpose, we split the array of weights w_{ij} into 20 sub-vectors of length Γ (i.e., a row if w_{ij} is reshaped to a $20 \times \Gamma$ matrix), each one related to a specific amino acid. To extract the most relevant linear combinations of coordinates in the Γ-dimensional space of sub-vectors in a group, we perform a principal component analysis (PCA)[6] on them. The PCA ranks the most relevant and independent linear combinations of coordinates in the Γ-dimensional space.

S2 STRIDE vs DSSP

It is important to notice that the location of a secondary structure's starting/ending point in proteins may depend on the algorithm chosen to detect them. In the main text, we show the results obtained with the DSSP. In this section, for comparison, we show the weights of an ensemble of RBMs trained with a database of α-helices obtained with the algorithm STRIDE[7] (see Figures S2, S3). The patterns observed do not deviate significantly from those shown in the main text for the DSSP (see Figures 4 and 5), indicating that the method based on the ensemble RBMs is sufficiently robust with respect to possible (small) deviations in the assignment of the extremities of secondary structures by different algorithms.

S3 Correlation matrices

Correlations between the occurrence of amino acids (say a, b) at different positions ($1 \leq i < j \leq \Gamma$) require the computation and parallel visualization of many matrices (one for every i-j pair). This, for example, is shown in Figure S4 for the start of α-helices. Each matrix element is

$$C_{ab}^{ij} = \langle I(a,i) I(b,j) \rangle - \langle I(a,i) \rangle \langle I(b,j) \rangle,$$

where $I(a,i) = 1$ if the amino acid at position i is a, and $I(a,i) = 0$ otherwise, and $\langle \ldots \rangle$ is the mean over the ensemble.

Of course, the correlations displayed in Figure S4 (and Figures S5, S6, and S7 for the other portions of the secondary structure) provide useful information. However, interpreting these results a priori, without the knowledge acquired by studying the RBMs' weights, seems more complicated.

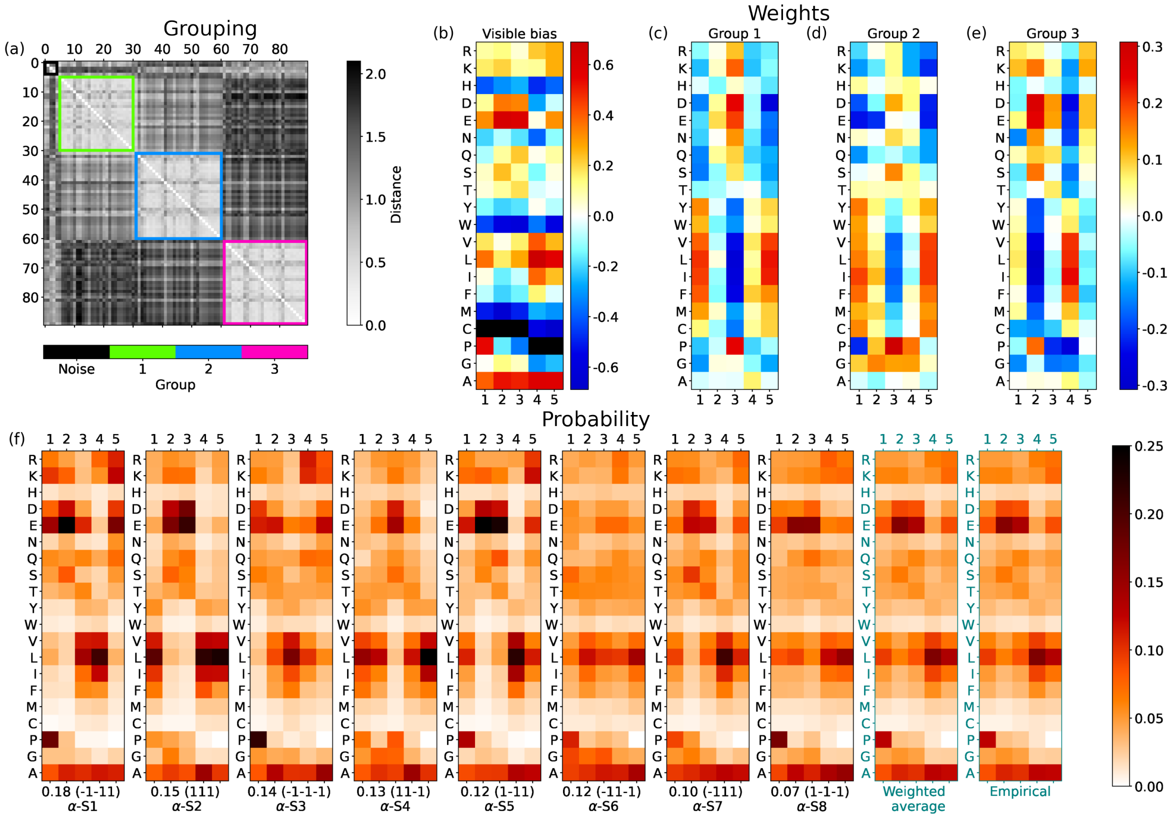

Figure S2: Results for the start of α-helices found with the algorithm STRIDE. See the legend of Figure 4 in the main text for more details.

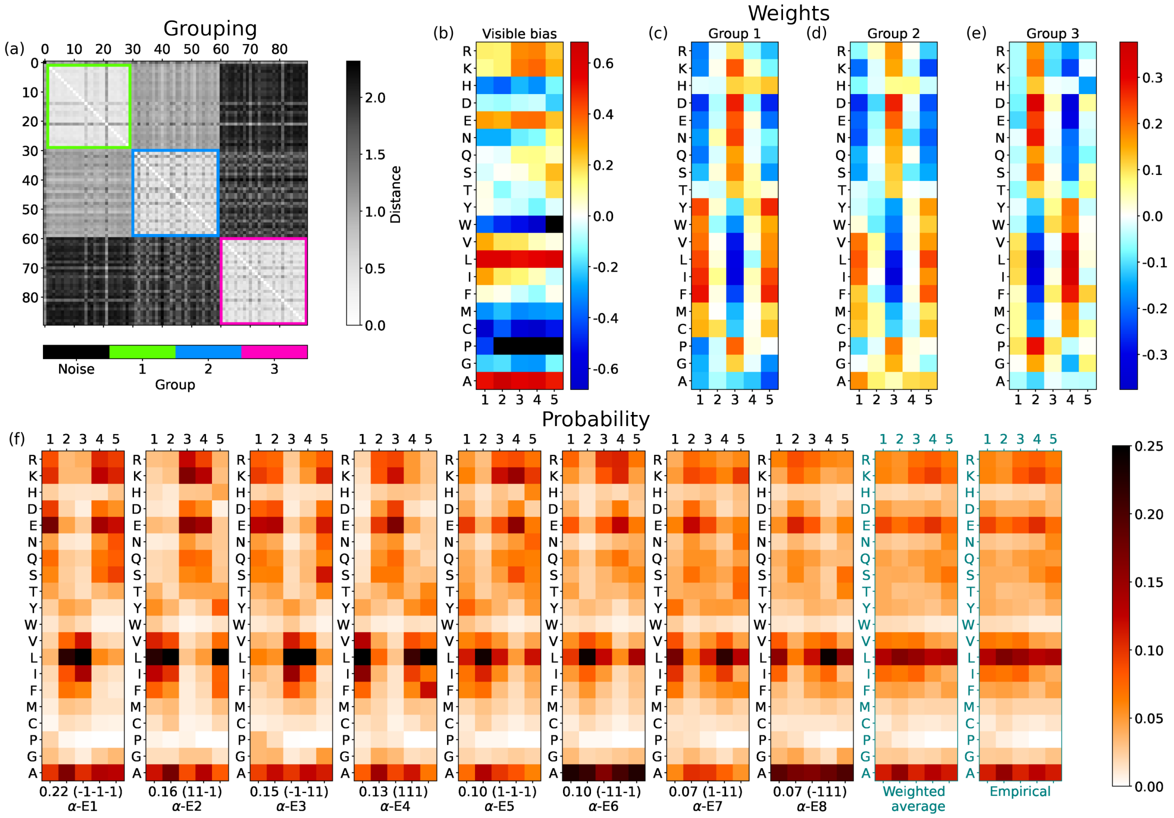

Figure S3: Results for the end of α-helices found with the algorithm STRIDE. See the legend of Figure 4 in the main text for more details. Compare this to Figure 5 in the main text.

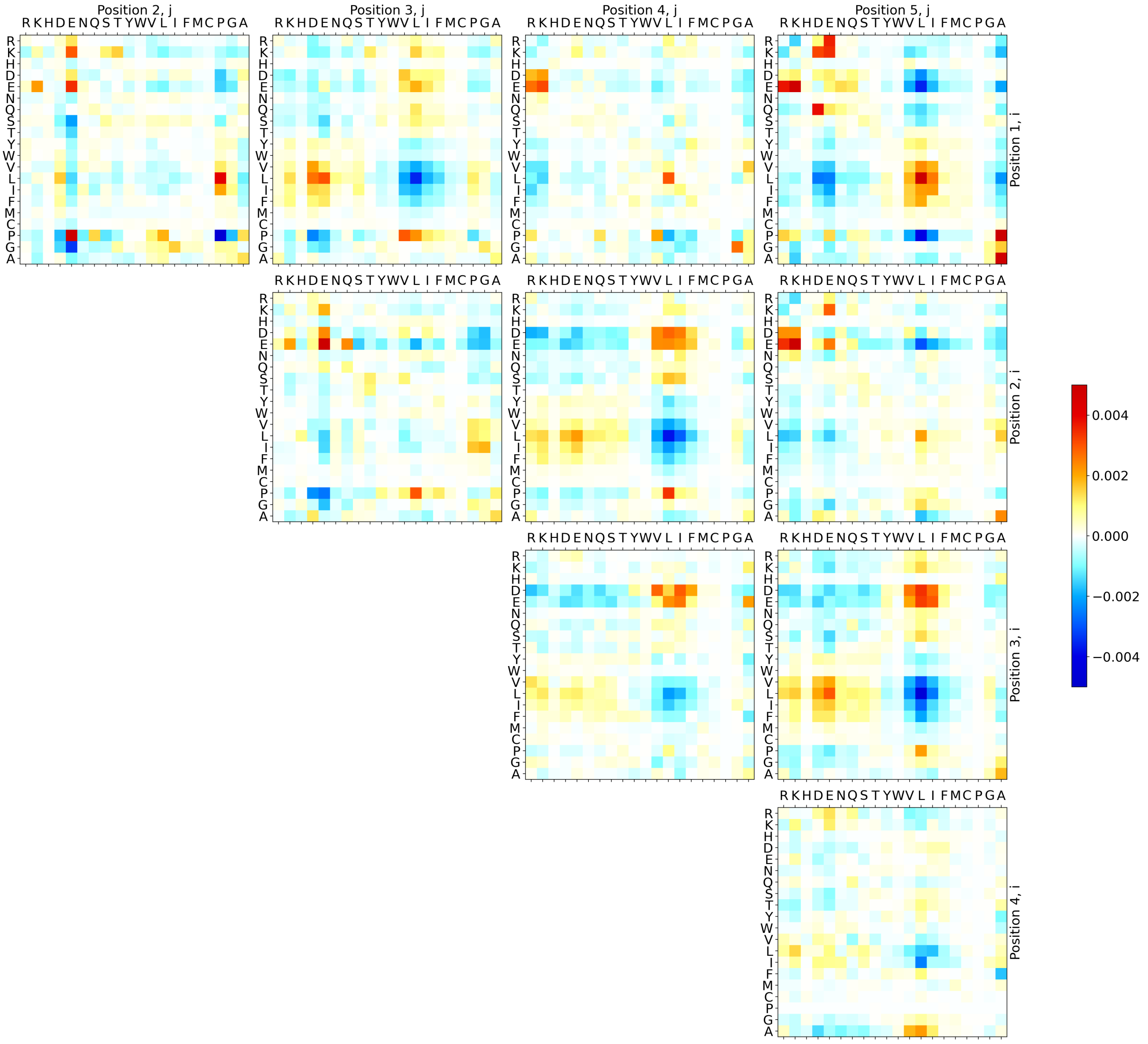

Figure S4: Covariance matrices C_{ab}^{ij} for the empirical occurrence of amino acid a at position i and amino acid b at position $j > i$. Each matrix is for a given i, j pair for the start of α-helices.

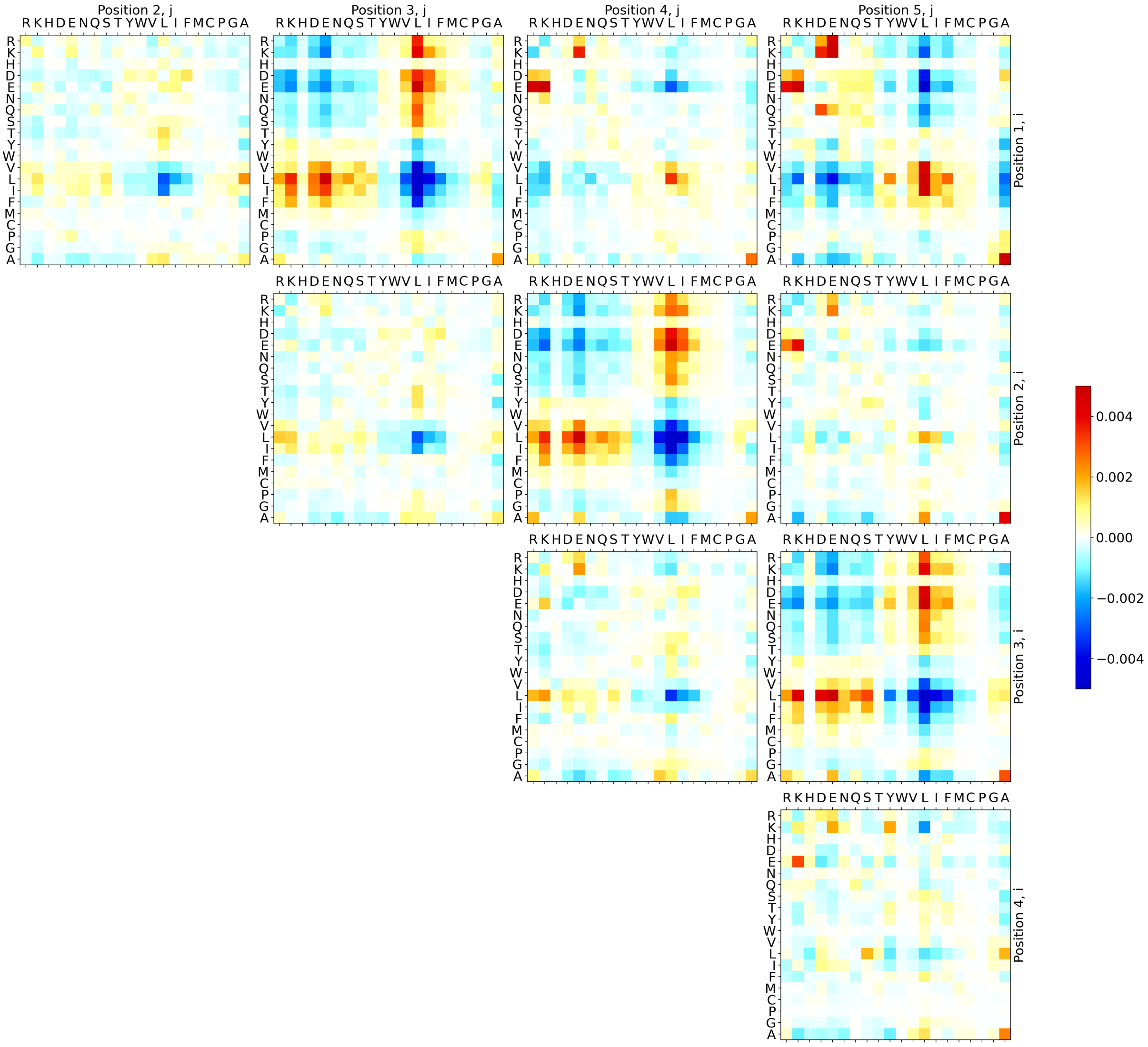

Figure S5: As in Fig. S4 but for the end of α-helices.

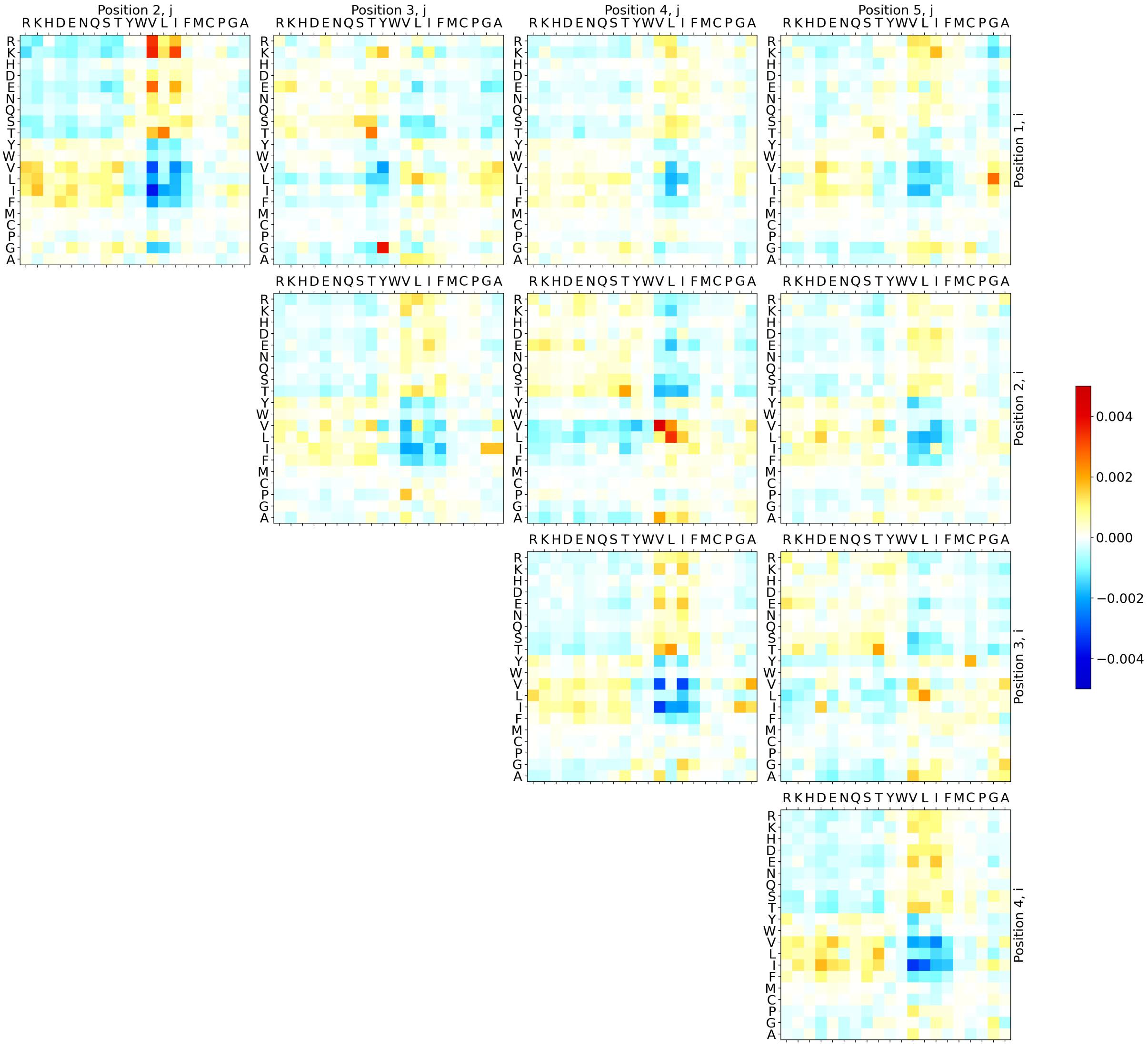

Figure S6: As in Fig. S4 but for the start of β-sheets.

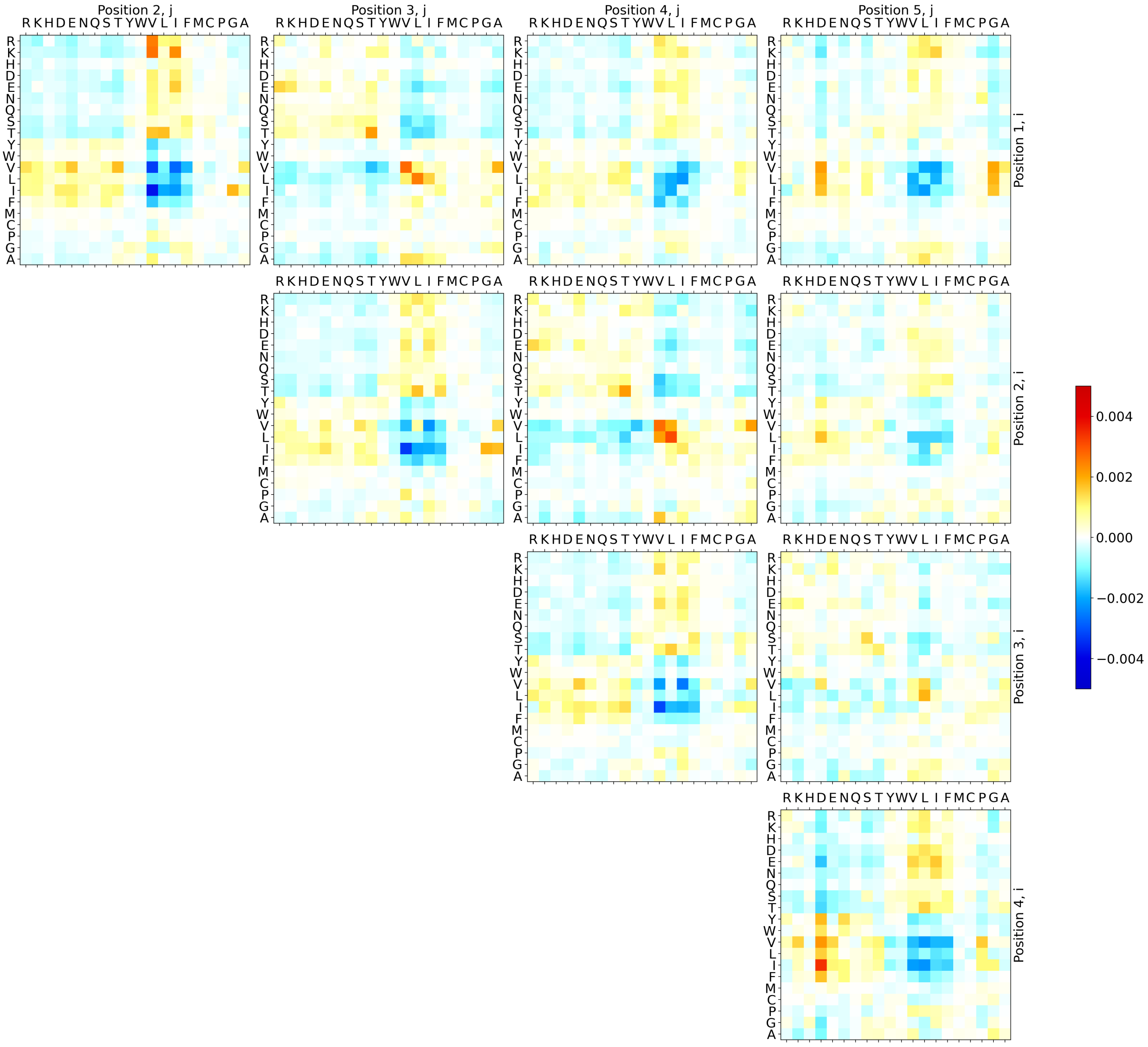

Figure S7: As in Fig. S4 but for the end of β-sheets.

References

(1) Hinton, G. E. *Neural networks: Tricks of the trade*; Springer, 2012; pp 599–619.

(2) Fischer, A.; Igel, C. Training restricted Boltzmann machines: An introduction. *Pattern Recognition* **2014**, *47*, 25–39.

(3) Tang, Y.; Sutskever, I. Data normalization in the learning of restricted Boltzmann machines. *Department of Computer Science, University of Toronto, Technical Report UTML-TR-11-2* **2011**, 27–41.

(4) Montavon, G.; Müller, K.-R. *Neural networks: tricks of the trade*; Springer, 2012; pp 621–637.

(5) Birant, D.; Kut, A. ST-DBSCAN: An algorithm for clustering spatial–temporal data. *Data & knowledge engineering* **2007**, *60*, 208–221.

(6) Mehta, P.; Bukov, M.; Wang, C.-H.; Day, A. G.; Richardson, C.; Fisher, C. K.; Schwab, D. J. A high-bias, low-variance introduction to machine learning for physicists. *Physics Reports* **2019**, *810*, 1–124.

(7) Frishman, D.; Argos, P. Knowledge-based protein secondary structure assignment. *Proteins: Structure, Function, and Bioinformatics* **1995**, *23*, 566–579.
